# A Mobility-Focused Knowledge Translation Randomized Controlled Trial to Improve Physical Activity: Process Evaluation of the Move4Age Study

**DOI:** 10.2196/13965

**Published:** 2019-06-20

**Authors:** Sarah E Neil-Sztramko, Jenna Smith-Turchyn, Julie Richardson, Maureen Dobbins

**Affiliations:** 1 School of Nursing McMaster University Hamilton, ON Canada; 2 Faculty of Kinesiology & Physical Education University of Toronto Toronto, ON Canada; 3 School of Rehabilitation Science McMaster University Hamilton, ON Canada; 4 National Collaborating Centre for Methods and Tools School of Nursing McMaster University Hamilton, ON Canada

**Keywords:** process evaluation, physical activity, health information, mobility, older adults, knowledge translation

## Abstract

**Background:**

Maintaining physical activity and physical function is important for healthy aging. We recently completed a randomized controlled trial of a targeted knowledge translation (KT) intervention delivered through the McMaster Optimal Aging Portal with the goal to increase physical activity and physical mobility in middle-aged and older adults, with results reported elsewhere.

**Objective:**

The purpose of this process evaluation study is to explore which KT strategies were used by both intervention and control group participants, as well as the intervention groups’ engagement, satisfaction, and perceived usefulness of the targeted KT intervention.

**Methods:**

Data on engagement with the intervention materials were gathered quantitatively through Google Analytics and Hootsuite throughout the intervention. Qualitative data were collected through a combination of open-ended surveys and qualitative interviews with a subset of participants at the end of the study to further understand engagement, satisfaction, and usefulness of the KT strategies.

**Results:**

Throughout the intervention period, engagement with content delivered through weekly emails was highest, and participants rated email content most favorably in both surveys and interviews. Participants were generally satisfied with the intervention, noting the ease of participating and the distillation of information in an easy-to-access format being beneficial features. Participants who did not find the intervention useful were those with already high levels of baseline physical activity or physical function and those who were looking for more specific or individualized content.

**Conclusions:**

This process evaluation provides insight into our randomized controlled trial findings and provides information that can be used to improve future online KT interventions.

**Trial Registration:**

ClinicalTrials.gov NCT02947230; https://clinicaltrials.gov/ct2/show/nct02947230 (Archived by WebCite at http://www.webcitation.org/78t4tR8tM)

## Introduction

Maintaining physical mobility is important for healthy aging and maintaining functional independence [1]. Regular physical activity has been shown to slow the age-related decline in physical mobility [2] and is associated with decreased risk of mobility disability and mortality [1,3,4]. Participation in regular physical activity has been shown to have a number of positive effects on functional, metabolic, cardiovascular, and cognitive outcomes, as well as improvements in quality of life [2]. Given these beneficial effects of exercise, effective knowledge translation (KT) interventions that disseminate information to middle-aged and older adults on strategies that promote physical mobility are warranted.

Population-based survey data from Canada suggest that older adults do use the Internet to seek out health information [5]. One potential benefit of health resources disseminated online is the potential for a wide reach to a diverse audience. However, it may be difficult for members of the public to adequately identify trustworthy online resources [6]. In response to this problem, the McMaster Optimal Aging Portal (the Portal) was created in 2014 to serve as a publicly available repository of high-quality, evidence-based information about healthy aging [7,8]. User characteristics [9] and usability [10,11] of the Portal have been previously reported. While it is clear that people are using the Portal, questions remain as to whether use of the Portal has any impact on knowledge, health behaviors, or health outcomes.

Recent systematic reviews suggest that electronic behavior change interventions may have positive effects on physical activity and other health outcomes [12,13]. A review of information technology-based interventions on patient engagement and behavior change found that while IT platform-based interventions had positive effects on health outcomes, there are few published reports of process data on engagement and usability within the interventions [12].

Our team recently completed a randomized controlled trial of a targeted KT intervention through the Portal, aimed at improving physical activity and physical mobility in middle-aged and older adults (to be published elsewhere, currently under review). In line with previously published recommendations on process evaluations alongside randomized controlled trials [14], the purpose of this process evaluation was to explore participants’ engagement, satisfaction, and perceived usefulness of the tailored KT intervention and the Portal in general. This provided a more in-depth understanding of our quantitative trial findings.

## Methods

### Trial Description

A full description of methods and results for this randomized controlled trial will be reported elsewhere. The Hamilton Integrated Research Ethics Board approved the study protocol, and all participants provided written informed consent. In brief, 510 participants—primarily female (430/510, 84.3%); mean age 64.7 years (SD 8.3; see Table 1)—were recruited online and randomized to a targeted KT intervention or self-serve control group. Approximately one-third were regular users of the Portal before the study, while one-third had never heard of the Portal before. Questionnaires were completed using an online survey platform at baseline (ie, prior to randomization), at the end of the 12-week study, and at 3-months postintervention. While there were no differences at the end-of-study or follow-up time points between groups for physical activity or self-monitoring, the intervention group did report more positive intentions to engage in mobility-related health behaviors and more favorable attitudes toward physical activity than the control group. In planned subgroup analyses to explore the effect of the intervention by participant characteristics, there was a significant intervention effect found among participants with low self-rated health.

### Intervention Description

During the intervention period, those in the intervention group received a targeted intervention consisting of the following:

1. Mobility-focused weekly emails, which included links to blog posts (ie, short articles providing recent scientific evidence in a narrative format), evidence summaries (ie, 1-2-page documents describing findings from a systematic review in lay language), and Web-resource ratings (ie, evaluations to assess quality of existing third-party websites) on a weekly topic related to physical activity and/or mobility.
2. Social media posts via Twitter and Facebook, using the study-specific hashtag #Move4Age to highlight relevant information related to physical activity or mobility. Participants were initially invited to follow social media feeds at the beginning of the intervention and were reminded throughout the intervention period via email.
3. Invitation to visit the *Mobility and Physical Functioning* page on the Portal.

As the Portal is a publicly available website, control group participants were able to access the Portal during the intervention period, including the Portal’s general weekly email alert subscription service; thus, our study did not include a *true* control group. The control group participants did not receive the targeted KT strategies, as described above.

**Figure 1:**
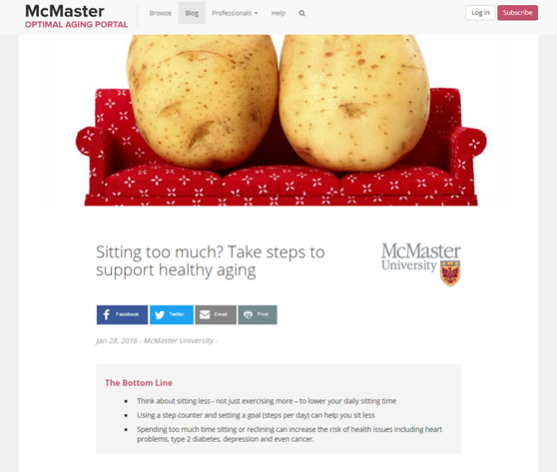
Example of intervention material delivered through the McMaster Optimal Aging Portal blog.

The targeted KT intervention was informed by the theory of planned behavior [15]. The theory of planned behavior suggests that intention to engage in a particular behavior is an immediate precursor of the behavior, and that intention is based on attitude toward the behavior, subjective norms, and perceived behavior control. Through this intervention, we aimed to modify individuals’ attitudinal beliefs through the provision of high-quality, evidence-based information about increasing physical activity and improving mobility and physical function. The educational materials provided were targeted at middle-aged and older adults and included actionable messages within the content, specifically within the blog posts [16], to act on normative and control beliefs (see Figure 1).

**Table 1:** Participant characteristics.

Characteristics		Total (N=510)	Intervention (n=256)	Control (n=254)	*P* value
Age (years), mean (SD)		64.7 (8.3)	64.7 (8.5)	64.6 (8.2)	.94
**Gender, n (%)**					.69
	Male	80 (15.7)	38 (14.8)	42 (16.5)	
	Female	430 (84.3)	218 (85.2)	212 (83.5)	
**Education, n (%)**					.91
	High school diploma or less	36 (7.1)	18 (7.0)	18 (7.1)	
	College diploma	111 (22.0)	58 (23.1)	53 (20.9)	
	Bachelor’s degree	217 (43.1)	104 (41.4)	113 (44.7)	
	Postgraduate degree	140 (27.8)	71 (28.3)	69 (27.3)	
**Employment status, n (%)**					.19
	Retired	304 (59.7)	157 (61.6)	147 (57.9)	
	Full-time employment	121 (23.8)	60 (23.5)	61 (24.0)	
	Part-time employment	65 (12.8)	28 (11.0)	37 (14.6)	
	Long-term disability	6 (1.2)	1 (0.4)	5 (2.0)	
	Other	13 (2.6)	9 (3.5)	4 (1.6)	
**Geography, n (%)**					.55
	Urban	422 (82.7)	209 (81.6)	213 (83.9)	
	Rural	74 (14.5)	41 (16.0)	33 (13.0)	
	Not reported	14 (2.7)	6 (2.3)	8 (3.1)	
Self-rated health: *Excellent* or *Very Good*, n (%)		303 (59.4)	144 (56.3)	159 (62.6)	.07
Chronic disease, n (%)		283 (55.7)	141 (55.3)	142 (56.1)	.92
**Falls**					
	Had a fall in the last 6 months, n (%)	103 (20.2)	41 (16.0)	62 (24.4)	.02
	Number of falls, mean (SD)	1.6 (1.2)	1.4 (0.9)	1.7 (1.3)	.19
	Visited a health care provider because of a fall, n (%)	35 (33.3)	15 (36.6)	20 (31.2)	.72
**Previous Portal^a^ use, n (%)**					.98
	Never used	172 (33.8)	87 (34.0)	85 (33.6)	
	Regular user	153 (30.1)	76 (29.7)	77 (30.4)	
	Used occasionally	184 (36.1)	93 (36.3)	91 (36.0)	
Sought information about improving mobility from a health care provider or other source in the last year, n (%)		220 (43.1)	118 (46.1)	102 (40.2)	.21

^a^Portal: McMaster Optimal Aging Portal.

### Data Collection

Data on engagement with intervention materials delivered through the mobility-focused weekly emails were collected from participants in the intervention group only. A custom campaign was created for each week of the intervention using Google Analytics. This provided data for each weekly email on the number of participants who opened a link within the email, number of website sessions, number of page views, number of pages viewed per session, time per session, and bounce rate (ie, the proportion of individuals who only viewed one page per session).

Intervention materials disseminated through social media (ie, Twitter and Facebook) were identified using a study-specific hashtag, #Move4Age, and thus were publicly available. Number of shares, likes, and links clicked (Facebook) as well as number of retweets, likes, and URL clicks (Twitter) were collected using Hootsuite (Hootsuite Inc).

Data on participant satisfaction with, and perceived usefulness of, the KT strategies for the intervention group only, and the Portal itself for both groups, were collected at the end-of-study and 3-month follow-up time points using a combination of Likert scales ranging from 1 (strongly disagree) to 7 (strongly agree) and open-ended questionnaires. A subgroup of 50 participants also consented to take part in qualitative interviews following the end-of-study data collection. Semistructured interviews were conducted by a trained interviewer who was not involved in any other aspect of the study. Interviews were recorded and transcribed verbatim.

### Data Analysis

Data on participant engagement with intervention materials is presented descriptively as mean and standard deviation as well as frequency and percentage where appropriate. Perceived satisfaction and usefulness of the Portal was compared between the intervention and control groups using an independent-samples *t* test for continuous variables and a chi-square test for categorical variables using SAS 9.4 (SAS Institute Inc).

Qualitative data from interview transcripts was entered into NVivo 11 (QSR International) for storage, indexing, searching, and coding. Two researchers (SENS and JST) reviewed a subset of interviews in duplicate to reach consensus on a coding scheme. Once agreement was reached, a thematic analysis was undertaken by the two researchers independently. Emergent themes were compared to open-ended survey questions and quantitative study results to provide a deeper understanding of our quantitative study findings.

## Results

### Engagement and Satisfaction With Knowledge Translation Strategies

**Figure 2:**
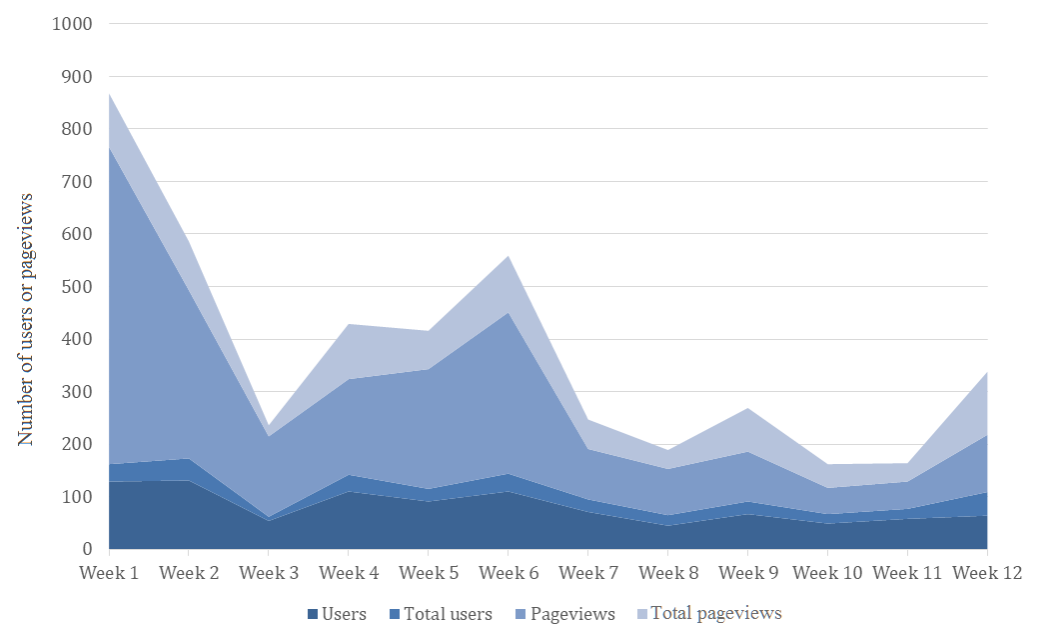
Intervention participant engagement with email content during the 12-week study period.

During the intervention period, 94.7% of intervention participants (198/209) reported receiving mobility-focused weekly emails. Engagement with email content was highest at the beginning of the study and declined throughout the course of the 12-week intervention (see Figure 2). Due to a technical issue, data on the number of emails successfully delivered and opened was not available. On average, one-third of intervention participants clicked through to the Portal from an email each week, ranging from a low of 17.6% in week 8 to 51.2% in week 2. Overall, engagement was highest in week 1 (766 total page views with an average of 4.07 pages and 5 minutes 32 seconds per session) and lowest in week 10 (117 total page views, 1.71 pages per session, and 1 minute 30 seconds per session). An increase in engagement was seen in the last week of the study: 218 total page views, 2.6 pages per session, and 2 minutes 36 seconds per session (see Table 2).

In qualitative interviews, participants reported that emails were the primary source of information utilized during the intervention period. With respect to positive aspects of the targeted KT intervention, a common theme emerged related to the ease of access to the study information. Participants reported that automatically receiving the mobility-focused content in their email inbox and having the large amount of information distilled in an easy-to-read format was appreciated.

I liked that it was information that came to me proactively. So I didn’t have to always go looking for it. It was also in bite-sized chunks. It was information that came in like, in sort of manageable pieces of time and information, and they offered you skills that you could develop pretty easily and quickly, it wasn’t a whole program you had to undertake, and it wasn’t, it was sort of easy pieces to fit into my life.

A related theme emerged around ease of selecting relevant content. Participants discussed selecting particularly relevant topics to read through, rather than reading through all information sent, which was viewed as a positive aspect of the intervention.

[My mother] is 84, I am 53, so I mean I look at my e-mail quite often, so, you know, I could see it, I knew it was there...sometimes I liked looking at the headings of what the evidence was, and you know I would peruse through and, you know, sometimes it was interesting to me, sometimes it was not—you know, but that was fine. I just deleted it, it was easy to delete if I wasn’t that interested in it.

**Table 2:** Intervention group engagement with mobility-focused email alerts during the 12-week intervention period.

Week	Topic	Unique users (N=256), n (%)	Total sessions, n	Total page views, n	Pages per session, n	Time per session, minutes, seconds	Bounce rate, %
1	Introduction to Move4Age	129 (50.4)	188	766	4.1	5, 32	35.1
2	Walking	131 (51.2)	209	493	2.5	3, 42	42.4
3	Enhancing social support with walking groups	54 (21.1)	78	215	2.5	2, 31	49.1
4	Balance	110 (43.0)	136	324	2.4	3, 8	42.9
5	Strength training	91 (35.5)	135	343	2.5	2, 55	43.4
6	Falls and injury prevention	110 (43.0)	152	451	2.9	3, 0	44.9
7	Maintaining a healthy body weight	71 (27.7)	95	191	2.2	3, 13	50.4
8	Using technology for self-monitoring	45 (17.6)	69	153	2.4	2, 13	51.4
9	Reducing sedentary time	67 (26.2)	86	186	1.0	1, 24	56.0
10	Alternative forms of exercise for mobility	49 (19.1)	63	117	1.7	1, 30	60.0
11	Cognition and mobility	58 (22.7)	81	129	1.7	1, 33	67.6
12	Overcoming physical limitations	64 (25.0)	85	218	2.5	2, 36	37.7
All weeks, mean (SD)		83.2 (30.8)	114.8 (46.4)	298.8 (183.2)	2.4 (0.7)	2, 46 (1, 5)	48.4 (9.0)

Only 7.7% (16/209) of intervention group participants reported using Twitter and 19.6% (41/209) reported using Facebook. Data on social media engagement by week is displayed in Table 3. During the study period, there were a total of 50 tweets marked with #Move4Age, ranging from 1 to 7 per week. These tweets garnered a total of 25,006 impressions (187-1485 impressions per tweet), 100 retweets (0-10 per tweet), 96 likes (0-10 per tweet), and 217 URL clicks (0-17 per tweet). The highest level of engagement was during weeks 3 and 4. The total number of study-specific Facebook posts was 15 (0-2 per week). These posts garnered 23,635 unique post impressions (278-3532 per post), 158 shares (1-28 per post), 298 likes (3-48 per post), 16 comments (1-4 per post), and 1206 link clicks (6-369 per post). The highest level of engagement was during weeks 1 and 6.

**Table 3:** Social media engagement throughout the 12-week study period.

Week	Twitter engagement						Facebook engagement				
	Tweets, n	Impressions per tweet, mean	Retweets per tweet, mean	Likes per tweet, mean	URL clicks per tweet, mean	Posts, n		Impressions per post, mean	Shares per post, mean	Likes per post, mean	Comments per post, mean
1	2	726.0	2.0	2.5	7.5	2		2359.5	15.5	32.5	1.0
2	7	260.9	0.3	0.9	3.3	1		2457.0	16.0	32.0	2.0
3	5	426.4	2.0	1.4	5.0	2		1042.5	6.5	14.0	1.5
4	4	811.8	4.3	4.3	10.8	2		1174.0	2.5	11.5	1.0
5	3	505.3	3.0	2.3	3.7	1		278.0	2.0	3.0	0.0
6	6	608.0	2.2	1.7	2.7	2		3439.0	28.0	38.0	4.0
7	6	420.3	1.7	1.3	4.0	1		513.0	3.0	7.0	0.0
8	7	397.0	1.9	1.7	2.9	1		1596.0	13.5	24.5	2.0
9	2	718.0	2.5	3.0	7.0	1		604.0	5.0	13.0	1.0
10	4	695.3	3.5	2.8	3.5	0		N/A^a^	N/A	N/A	N/A
11	3	275.3	0.0	2.0	1.3	1		1845.0	10.0	13.0	1.0
12	1	841.0	3.0	1.0	8.0	1		2155.0	18.0	27.0	0.0
All weeks, mean (SD)	4.2 (2.0)	557.1 (202.9)	2.2 (1.2)	2.1 (1.0)	5.0 (2.8)	1.3 (0.6)		1587.5 (1035.0)	10.9 (8.4)	19.6 (12.5)	1.2 (1.2)

^a^N/A: not applicable; there were no Facebook posts in week 10.

### Control Group Engagement With the Portal

During the intervention period, 89.4% of control group participants reported registering for the Portal’s regular weekly email alert subscription service, 4.6% reported following the Portal on Twitter, 19.4% reported following the Portal on Facebook, and 29.6% reported browsing for mobility-related content on the Portal (see Table 4). There were no significant differences in engagement with the different strategies between intervention and control group participants. Fewer control group participants reported that information from the Portal influenced a decision they made about physical activity (54.5% vs 68.0%, *P*=.006). Of those that reported that the Portal did influence a decision, the control group reported that this occurred less often (mean 2.73, SD 1.90 vs mean 3.43, SD 2.06, where 1 is *not often* and 7 is *very often*, *P*<.001).

### Perceived Usefulness of the Intervention

At the end of study, participants reported mobility-focused emails to be useful (mean 5.27, SD 1.52, on a 1-7-score Likert scale) and reported favorably regarding their likelihood of continuing to subscribe (mean 5.46, SD 1.78) and recommending to a friend or family member (mean 5.29, SD 1.81; see Table 4). Of those who did use social media, responses were similarly favorable for usefulness of both Twitter and Facebook, likelihood of continued use, and likelihood of recommending to family or friends. The overall satisfaction with the intervention itself and with particular KT strategies was echoed by intervention group participants in qualitative interviews; however, a number of divergent themes emerged among participants with respect to the perceived usefulness and potential impact of the intervention. A first group of participants reported that they learned something new during the intervention that resulted in them making lifestyle changes around physical activity.

**Table 4:** Participant satisfaction and Portal^a^ use during the 12-week intervention period.

Portal activity and influence		Intervention (n=209)	Control (n=216)		*P* value
**Weekly email alerts from the Portal**				.06	
	Received weekly email alerts, n (%)	198 (94.7)	193 (89.4)		
	Mobility-specific email alerts are a useful strategy, mean (SD)	5.27 (1.52)^b^	N/A^c^		
	Would continue to subscribe, mean (SD)	5.46 (1.78)	N/A		
	Would recommend to a friend or family member, mean (SD)	5.29 (1.81)	N/A		
**Portal access via Twitter**				.27	
	Accessed the Portal via Twitter, n (%)	16 (7.7)	10 (4.6)		
	Twitter is a useful strategy, mean (SD)	5.07 (1.87)	N/A		
	Will continue to use, mean (SD)	6.12 (1.41)	N/A		
	Would recommend to a friend or family member, mean (SD)	5.56 (1.50)	N/A		
**Portal access via Facebook**				.99	
	Accessed the Portal via Facebook, n (%)	41 (19.6)	42 (19.4)		
	Facebook is a useful strategy, mean (SD)	5.61 (1.43)	N/A		
	Will continue to use, mean (SD)	5.90 (1.30)	N/A		
	Would recommend to a friend or family member, mean (SD)	5.32 (1.65)	N/A		
***Mobility and Physical Functioning* browse page**				.34	
	Used the *Mobility and Physical Functioning* browse page, n (%)	72 (34.4)	64 (29.6)		
	Mobility-specific browse page is a useful strategy, mean (SD)	5.60 (1.10)	N/A		
	Will continue to use, mean (SD)	5.51 (1.35)	N/A		
	Would recommend to a friend or family member, mean (SD)	5.30 (1.60)	N/A		
**Portal information influenced a decision about physical activity**					
	Number of participants who answered *yes*, n/N (%)^d^	140/206 (68.0)	116/213 (54.5)		.006
	How often? mean (SD)	3.43 (2.06)	2.73 (1.90)		<.001

^a^Portal: McMaster Optimal Aging Portal.

^b^Numerical questions were answered on a scale of 1 (not often) to 7 (very often).

^c^N/A: not applicable.

^d^There were missing data (n=6) from this question: intervention (n=3) and control (n=3).

Certainly, I have been upping my exercises that involve pounding but stress on the muscles and the bone- the reason swimming doesn't do it because you have no impact. I certainly have continued to focus on impact activity.

I was not previously aware that I needed to walk faster than a pleasant stroll. Now I am aware and each night after dinner my husband and I walk a fast 25 minutes.

A second group of participants described the intervention materials as serving an important reminder to engage in health behaviors or reinforcement, but the content did not contain a lot of new information or result in new knowledge gained.

Yeah, in the sense that it just alerted me and kept me, kept me, ah, on target with my workouts and my walks, bicycling, and all that kind of stuff.

For example, bone density is an issue for myself and it really wasn't always new information. It really more confirmed what I always researched and found out. Whether I’ve used it or not...um, for example, you're not supposed to swim, for example, as it's not an activity that increases bone density and I think I read that somewhere, but I already knew that. I'm not a totally uninformed consumer.

A third group of participants noted that while they were satisfied with the intervention materials and content, they found the intervention not particularly impactful because they were already active or had no mobility limitations.

Yeah, not for any reason, ah, I’m relatively mobile, relatively mobile myself, but, um, that becomes an issue as you age and I suppose it’s better to know about it before it’s an issue, so I found it very interesting.

You know, that again, I was pretty mobile, I had no, no issues beforehand, and I still don't have any issues, so although it didn't improve, it's because it would have been pretty hard for it to improve, I think.

A final group of participants reported that the intervention itself had no impact and that they were generally disappointed with the intervention. Two prominent subthemes emerged within this group. Firstly, participants were dissatisfied with the intervention because the information provided was not specific enough.

It didn't really...it was superficial. It didn't tell you what to do, where to go...it was kind of information that's out there everywhere. There was nothing really new. I read it a couple of times and thought I’m missing something here...and then at some point I stopped reading them because I thought I would just glance over it because I thought that, I want the meat, okay? I don't want any more of these studies and this here and that...nothing gets in my pocket. My pocket meaning...I’m not getting any services.

Secondly, some participants reported that the intervention seemed more appropriate for individuals with lower levels of baseline health or fitness.

I had a sense that it was targeted at people with already quite limited mobility, and not including those who had perhaps re-achieved a higher level of mobility through their own initiative.

A lot of it seemed to be directed at people that had much more significant problems than me, so I’m pretty active and quite healthy, and so I was looking for things that would sort of help me stay that way or any tips, or any new information. And, yeah, I felt that a lot of the information, not all of it, but a lot of it was directed at people that already had significant problems.

## Discussion

Findings from our randomized controlled trial suggest that a targeted KT intervention may have a positive impact on levels of physical activity in middle-aged and older adults, particularly those with low baseline self-rated health. Findings from the process evaluation presented here help to understand these findings and provide guidance on the design and delivery of future KT interventions, particularly those using an online platform such as ours. To our knowledge, this is the first process evaluation conducted of an online KT intervention targeted at middle-aged and older adults.

Despite the large number of intervention participants who reported receiving the mobility-focused weekly emails, actual engagement with the Portal content as measured through Google Analytics was much lower. The proportion of participants in the intervention group who visited the Portal through a link within an email ranged from 17.6% to 51.2% (weeks 8 and 2, respectively). While lower than our target, these are still much higher than industry averages for health services email campaigns, which report average email open rates of 19.2% and click-through rates of only 6.4% [17]. In addition, those who did click through seem to be well engaged, which is reflected in the average number of pages viewed per session and the average time per session. The average length per session of 2 minutes and 46 seconds suggests that participants may have read the majority of the article on the page they visited. This hypothesis was confirmed in our qualitative interviews, where participants reported only clicking on links they were interested in reading and reading through the content on pages they visited. To our knowledge, few other similar studies have been conducted to provide comparative data. In a recent process evaluation of usage data from a publicly available, Web-based mental health portal for youth, 65.4% of sessions were less than one minute in duration [18]. In contrast, a Web-based intervention of interactive self-monitoring modules for women to prevent weight gain reported a median session duration of 12 minutes and 54 seconds [19]. However, it is difficult to directly compare session length, due to potential differences in the amount of content, education levels, and background knowledge of participants.

Qualitative data provided some explanation as to why such a low percentage of participants clicked through from each weekly email, despite rating the emails as highly useful. Participants reported that one of the benefits of receiving the weekly email alerts was that it allowed them to quickly and easily identify personally relevant and topical material to read more thoroughly. We hypothesize that the length per session is reflective of participants being more interested and engaged with the content to which they chose to click through from the original email, while individuals did not click through to content that they were less interested in. In a process evaluation of a sexual health website, average time on the site ranged from 2 minutes and 7 seconds to 6 minutes and 36 seconds, depending on keywords searched and the referring website, demonstrating the substantial variability in usage that can occur within the same site depending on the topic at hand [20]. The intervention period did not occur over any major holidays, and we cannot identify any external reasons that would contribute to increased uptake in particular weeks (eg, week 6). To minimize participant burden, we did not collect feedback on weekly intervention content, although this information would be useful in further understanding what drives weekly variation in intervention engagement.

Given that there were no significant differences found in the proportion of participants in both the intervention and control groups that reported receiving weekly email alerts, following the Portal on social media, and following the mobility landing page, it is not surprising that no significant between-group differences were found at the end-of-study or postintervention follow-up data collection. Our findings that both groups self-reported significant increases in physical activity and self-monitoring mobility over time may indicate that the Portal’s currently available KT strategies are sufficient to elicit behavior change, at least in some individuals. However, given the variation in change in physical activity across participants, and the still low proportion of participants meeting physical activity guidelines at the end of the study, it may also be that more specific tailoring of intervention materials is needed to see greater behavior change in some individuals.

As discussed above, a major theme that emerged from qualitative interviews was the benefit of ease of access to content through the weekly email alerts, and the *filtering* of relevant information. It may be that control group participants who signed up for this study due to their interest in physical activity and mobility also filtered the general Portal content in the same way. We were not able to track usage data in the form of number of clicks in email alerts through the control group, so we are not able to evaluate whether there was a difference in engagement between the targeted intervention email alerts and the general Portal email alerts to confirm this hypothesis.

Conversely, we also identified a group of participants within the qualitative interviews who were dissatisfied with the intervention due to its focus on general information and lack of specific instruction or resources. These findings are similar to those from our previous cross-sectional survey of Portal users, where a thematic analysis of open-ended questions identified limitations of the Portal being that information was not specific or in-depth enough [9]. This is an ongoing challenge for those designing online resources and delivering online KT interventions. These online resources must balance the logistics and feasibility of disseminating a broad-reaching behavior change intervention, while also being relevant to an individual. In a recent systematic review of eHealth interventions for physical activity promotion in older adults, four of the six website-based studies found a significant increase in physical activity compared to a no-intervention control group [13]. Each of these studies used some type of interactive component with intervention participants, such as a personal coach [21], an interactive website feature to track physical activity [22,23], or provision of a pedometer or accelerometer for monitoring physical activity [23,24]. While found to be effective, these components may not be feasible on a large-scale, publicly available resource such as the Portal. In a systematic review of strategies to facilitate use of Internet-delivered, health behavior change interventions in younger adults, Crutzen et al found that tailored communications, reminders, and incentives are useful strategies to increase user engagement with intervention content [25]. Qualitative data from our participants highlighting the greater engagement with personally relevant content support the use of tailoring in delivering intervention content. Future work may explore whether more specific tailoring of the intervention materials provided, such as by baseline values, knowledge, or preferences, may elicit greater overall behavior change and satisfaction with the KT intervention. Perhaps a theory-driven approach to tailoring, for example, using the Transtheoretical Model, could be explored to tailor intervention material based on an individual’s stage of change [26].

Qualitative interviews identified a subgroup of participants who were satisfied with the intervention but did not report it being particularly impactful because of their already high levels of baseline health. This is in line with findings from our quantitative subgroup analysis from our recently completed randomized controlled trial, in that there was a significant effect of the intervention on physical activity levels of those with poor or fair baseline self-rated health. Measures of self-rated health have been found to be correlated with perceived physical fitness [27], physical mobility [28], and mortality [27,29]. Although baseline physical activity levels were controlled for in the analysis, those with the highest self-rated health at baseline may have been the most healthy and active; thus, change in physical activity throughout the intervention period may be limited by a ceiling effect, as suggested by several participants within the qualitative interviews. Interestingly, the percentage of participants who met physical activity guidelines was 27.4% in the intervention group and 29.4% in the control group at baseline. We hypothesize that individuals perhaps perceived they were sufficiently active and thus did not need to increase their physical activity levels, which contributed to the lack of change. Future interventions should consider including a feedback mechanism to bring awareness to participants’ reported or measured physical activity levels prior to the start of the intervention.

A limitation to this process evaluation, and to our understanding of how engagement with the intervention materials correlated to behavior change, is our inability to access Google Analytics data on clicks per link from individual participants. We hypothesize that those who engaged most with the intervention materials may be most likely to have changed their behavior as a result of the intervention, however, we were not able to evaluate this, given the aggregate group-level data. Our sample was primarily well-educated females; thus, our findings may be less applicable to online portals targeting other populations. In addition, all social media posts and hashtags were publicly available, so we are unable to attribute any tracked engagement exclusively to participants in our intervention group.

We have previously reported on the significant increase in physical activity levels observed in both groups and, in particular, the significant intervention effect in participants with low self-rated health at baseline. When combined with positive findings in this process evaluation on participant satisfaction and engagement with the intervention strategies and the Portal itself, we believe KT strategies such as those delivered through the Portal have the potential to be an effective, low-cost, and scalable intervention. Insights from this process evaluation suggest that targeting the appropriate population is an important consideration. Delivering the intervention to individuals with the greatest need (ie, those with low self-rated health) or to those with the greatest potential to show a change (ie, low baseline levels of physical activity) should be explored in future studies. Previous research has shown that individuals who use online health portals are typically more highly educated and have higher health literacy [30]. A challenge in conducting this work is to understand how to engage underserved groups in an online intervention such as ours. Further work is needed to understand which KT strategies may be most effective to increase knowledge, awareness, and engagement with a resource such as the Portal.

## Abbreviations

KT: knowledge translation
Portal: McMaster Optimal Aging Portal
